# General versus general anaesthesia combined with caudal block in laparoscopic-assisted Soave pull-through of Hirschsprung disease: a retrospective study

**DOI:** 10.1186/s12871-021-01431-5

**Published:** 2021-08-30

**Authors:** Zhixiong Lin, Yifan Fang, Lei Yan, Yu Lin, Mingkun Liu, Bing Zhang, Yuanbing He, Yong Shen, Dianming Wu, Longxin Zhang

**Affiliations:** 1 Department of Pediatric Surgery, Fujian Maternity and Child Health Hospital, Affiliated Hospital of Fujian Medical University, Fujian, China; 2 Department of Anesthesiology, Fujian Maternity and Child Health Hospital, Affiliated Hospital of Fujian Medical University, No.18 Daoshan Road, Fuzhou, 350001 Fujian China

**Keywords:** Caudal block, Hirschsprung disease, General anesthesia

## Abstract

**Background:**

Caudal block is one of the most preferred regional anesthesia for sub-umbilical region surgeries in the pediatric population. However, few studies are available on caudal block performed in laparoscopic-assisted Soave pull-through of Hirschsprung disease (HD). We aimed to compare general anesthesia (GA) and general anesthesia combined with caudal block (GA + CA) in laparoscopic-assisted Soave pull-through of HD.

**Methods:**

A retrospective review was performed in children with HD operated in our hospital between 2017 and 2020. Patients were divided into the GA and GA + CA group. The primary outcome was the duration of operation, and secondary outcomes included intraoperative hemodynamic changes, the Face, Legs, Activity, Cry, Consolability (FLACC) scale, dose of anesthetics, and incidence of side effects.

**Results:**

A total of 47 children with HD were included in the study, including 20 in the GA group and 27 in the GA + CA Group. The two groups were similar in age, gender, weight and type of HD (*P* > 0.05). The GA + CA group had significantly shorter duration of operation (especially the transanal operation time) (median 1.20 h vs. 0.83 h, *P* < 0.01) and recovery time (mean 18.05 min vs. 11.89 min, *P* < 0.01). The mean doses of sufentanil and rocuronium bromide during the procedure and FLACC scores at 1 h and 6 h after surgery were also lower in the GA + CA group (*p* < 0.01). The hemodynamic changes in the GA + CA group were more stable at time of t_2_ (during transanal operation) and t_3_ (10 min after transanal operation), but there was no significant difference in the incidence of postoperative side effects between the two groups (*P* = 1.000).

**Conclusion:**

General anesthesia combined with caudal block can shorten the duration of operation, and provide more stable intraoperative hemodynamics and better postoperative analgesia.

## Background

Hirschsprung disease(HD) is a common congenital malformation of the digestive tract in infants which characterized by the absence of enteric neurons in the distal colon. The incidence of HD ranges from 1:3,500 to 1:10, 000 live births. Neonates presenting with delayed passage of meconium (> 48 h), abdominal distention, and emesis are suspected of HD and the diagnosis is confirmed before 6 months of age [1, 2]. Surgery is the primary treatment for HD, and we perform an averaged 25 operations in our hospital per year. Surgical treatment includes removing the intestine where the enteric neurons are missing, and reconstructing the digestive tract, which needs to be perfoemed through the anus. The anal canal is narrow and the internal sphincter is very weak in infants, making the pull-through procedure difficult. Excessive anal canal traction during operation can damage the internal sphincter and cause adverse effects [3]. Therefore, adequate analgesia and anal muscle relaxation induced by anesthesia are very important during the operation.

Caudal blocks is one of the most preferred regional anaesthesia for sub-umbilical region surgeries in the pediatric population [4], and its advantages include its simplicity, safety and low rate of complications. It has been widely used in pediatric surgery, such as hypospadias repair, circumcision and inguinal hernia repair [5, 6]. Caudal blocks can decrease the erethism of sympathetic nerves and have the asme obvious effects of analgesic and muscle relaxants, facilitating the operation and promoting postoperative recovery. At present, few studies are available on the administration of caudal block in HD. We hypothesized that general anesthesia combined with caudal block could effectively relax the anal muscles making it easy to pull out the colon through the muscular sheath, thus greatly reducing the difficulty of the operation and shortening is duration of operation. In this study, we evaluated the effect of caudal block on the duration of operation, analgesic use and postoperative pain intensity in laparoscopic-assisted Soave pull-through pediatric population referred to our hospitals.

## Methods

### Study design

A retrospective review was carried out on inpatient surgical records to identify children aged 3 to 6 months who underwent laparoscopic-assisted Soave pull-through procedures for HD between January 2017 and June 2020. According to the methods of anesthesia, they were divided into two groups: general anesthesia group (GA group) and general anesthesia combined with caudal block group (GA + CA group). The study protocol was approved by the ethics committee of the Fujian Maternity and Children Health Hospital (No.2020YJ227) before beginning the study.

#### Anesthesia protocol

The protocol for GA was the same for all patients, as follows: patients were induced with midazolam 10 μg/kg, sufentanil 0.4 μg/kg, propofol 2 mg/kg and rocuronium 0.6 mg/kg. Anesthesia was maintained using sevoflurane at 1.0 to 1.2 minimum alveolar concentration (MAC) and a combination of 30%—40% oxygen. GA + CA was performed with a single-injection of 1.0% lidocaine and 0.15% ropivacaine at a dose of 0.8 ml/kg. In both groups, administration of sufentanil was repeated at 0.1 μg/kg during surgery whenever the heart rate (HR) or blood pressure increased by more than 20% of their baseline levels. Rocuronium was added at a single dose of 0.2 mg/kg when peak inspiratory pressure (PIP) was > 20%. All children underwent laparoscopic-assisted Soave pull-through procedures performed by the same pediatric surgeon. The inhalation of sevoflurane was stopped 10 min before the end of the operation, and tracheal intubation was removed after the children's spontaneous breathing recovered.

### Evaluation of intraoperative hemodynamic, duration of operation, recovery time, transanal operation time, postoperative pain intensity and postoperative side effects

The data collected included patient gender, age, weight, type of HD, duration of operation, and dose of sufentanil and rocuronium bromide used during the procedure. The duration of the operation was measured from the placement of the first-stay suture to the end of the operation. Recovery time was defined as the time from the discontinuation of sevoflurane to spontaneous opening of the eyes. Transanal operation time was measured from the separation of the rectal sheath to completed the digestive tract reconstruction. Hemodynamic changes (including HR, systolic blood pressure (SBP) and diastolic blood pressure (DBP)) were recorded before anesthesia induction (t_0_), at the beginning of operation (t_1_), at the time of transanal operation (t_2_), and 10 min after transanal operation (t_3_). The intensity of postoperative pain was evaluated using the Face, Legs, Activity, Cry, Consolability (FLACC) scale [7], which scores pain intensity by rating five behaviours (face, legs, activity, consolability and cry) to derive a score range 0–10. Each item has a possible value of 0 to 2, with a maximum total FLACC score of 10 indicating maximum pain(0 = relaxed/comfortable, 1–3 = mild discomfort, 4–6 = moderate pain, 7–10 = severe discomfort/pain). The FLACC score was estimated at 1, 6, 12 and 24 h after surgery. The incidence of side effects after extubationincluding laryngospasm, restlessness, nausea and vomiting, were compared between the two groups.

### Statistical analysis

The sample size was calculated using the PASS software version 11: Two-Sample T-Test Power Analysis. According to a previous study [3], the mean duration of operation was 2.96 h with a standard deviation of 0.3 h, a sample size of 36 patients (18 in each group, 2 groups) was required to achieve a power of 80%, and a one-sided 95% confidence interval.

The data were analyzed using the SPSS 20.0 statistical software. Characteristics were compared between the two groups using Student's t-test for continuous variables and the chi-square test for categorical variables. When the expected counts were less than 5, Fisher's exact test was used. A *P*-value < 0.05 was considered statistically significant difference.

## Results

A total of 47 children who were diagnosed with HD by pathological biopsy and underwent laparoscopic-assisted Soave pull-through procedures were enrolled in this study, including 20 in the GA group and 27 in the GA + CA group. The demographics of the patients are shown in Table 1.

**Table 1:** Clinical characteristics of studied patients with HD underwent laparoscopic-assisted Soave pull-through procedures with and without caudal blocks

Variables	Group GA (n = 20)	Group GA + CA (n = 27)	*P*
Demographic characteristics			
Age, months, mean (range)	4.70(3.0, 5.9)	5.07(3.07, 6.00)	0.409
Gender (M/F)	16/4	24/3	0.438
Weight, kg, mean (range)	7.05(3.0, 9.7)	7.10(4.80, 8.50)	0.503
Type of HD			0.943
Short segment type n(%)	5(42%)	7(58%)	
Typical-segment type n (%)	15(43%)	20(57%)	
Duration of operation, h, median (range)	2.94(2.50, 3.85)	2.84(1.5, 3.4)	0.040
Transanal operation time, h, media (range)	1.20 (0.80, 1.67)	0.83 (0.5, 1.33)	< 0.01
Recovering time, min, mean (SD)	18.05 ± 4.9	11.89 ± 4.2	< 0.01
Dose of sufentanil use, µg/kg, mean (± SD)	0.39 ± 0.10	0.12 ± 0.09	< 0.01
Total dose of sufentanil use, µg, mean (± SD)	2.89 ± 1.11	0.90 ± 0.67	< 0.01
Dose of rocuronium bromide use, mg/kg, mean (± SD)	0.20 ± 0.08	0.07 ± 0.04	< 0.01
Total dose of rocuronium bromide use, mg, mean (± SD)	1.51 ± 0.69	0.58 ± 0.34	< 0.01

Note: Ages, weights and operating times are expressed as medians with range. SD; standard deviation

The groups were similar in their gender (*P* = 0.438) and HD type distribution (*P* = 0.943). The weight (median 7.05 kg GA vs.7.10 kg GA + CA; *P* = 0.503) and age at surgery (median age 4.70 months GA vs. 5.07 months GA + CA; *P* = 0.409) were also similar between the two groups. Compared with the GA group, the GA + CA group had significantly shorter duration of operation (median 2.94 h vs. 2.84 h, *P* = 0.040), and in particular significantly shorter duration of transanal operation (median 1.20 h vs. 0.83 h, *P* < 0.01). The recovery time after extubation in the GA + CA group was significantly shorter than that in the GA group (18.05 ± 4.9 min vs. 11.89 ± 4.2 min, *P* < 0.01). The mean doses of sufentanil and rocuronium bromide during the procedure were also lower in the GA + CA group ( *p* < 0.01) (Table 1).

Hemodynamic (HR, SBP and DBP) changes during the operation of the two groups were similar before intubation (t_0_) and at the beginning of operation (t_1_)(*P* > 0.05). At the time of transanal operation (t_2_) and 10 min after transanal operation (t_3_), the mean HR in GA + CA group was significantly lower than that in the GA group (130.95 ± 18.64 vs. 118.18 ± 14.29, *P* = 0.011 and 133.35 ± 18.61 vs. 119.62 ± 13.55, *P* = 0.005), but there were no significant differences in SBP or DBP between the two groups. Hemodynamic parameters during the operation in the GA + CA group were more stable than those in the GA group (Tables 2).

**Table 2:** Comparison of hemodynamic changes between the GA and GA + CA groups

Parameter	Time	Group GA (n = 20)	Group GA + CA (n = 27)	*p*
HR (mmHg), mean (± SD)	t_0_	135.35 ± 13.94	132.44 ± 14.71	0.497
	t_1_	150.00 ± 12.11	144.37 ± 13.58	0.149
	t_2_	130.95 ± 18.64	118.18 ± 14.29	0.011
	t_3_	133.35 ± 18.61	119.62 ± 13.55	0.005
SBP (mmHg), mean (± SD)	t_0_	92.15 ± 8.08	91.19 ± 7.43	0.674
	t_1_	80.30 ± 6.63	82.77 ± 5.83	0.181
	t_2_	81.10 ± 5.15	83.14 ± 6.09	0.231
	t_3_	80.65 ± 4.71	83.29 ± 6.00	0.110
DBP (mmHg), mean (± SD)	t_0_	53.20 ± 5.00	52.59 ± 4.90	0.679
	t_1_	51.60 ± 6.35	53.03 ± 6.49	0.453
	t_2_	52.15 ± 7.01	50.33 ± 6.51	0.371
	t_3_	49.65 ± 5.90	50.51 ± 6.48	0.640

SD; standard deviation; t_0_, time at preinduction just before administration of propofol; t_1_, at the moment of the surgical incision; t_2_, at the time of transanal operation; t_3_, 10 min after transanal operation

**Table 3:** Comparison of side effect after extubation between the two groups

Parameter	Group GA (n = 20)	Group GA + CA (n = 27)	*P*
Side effect n(%)	7(35%)	7(25.9%)	0.452
Laryngospasm n(%)	1(5%)	1(3.7%)	1.000
Move restlessly n(%)	3(15%)	2(7.4%)	0.638
Nausea and vomiting n(%)	3(15%)	4(14.8%)	1.000

There was no significant difference in the incidence of side effects (including laryngospasm, restlessness, nausea and vomiting) between the two groups (35% vs. 25.9%, *P* > 0.05) (Table 3). The FLACC scores used to assess pain and distress levels were significantly lower in the GA + CA group than in the GA group (1 h after operation: GA, 5.35 ± 1.22 vs. GA + CA 3.33 ± 1.00, *P* < 0.01; 6 h after operation: GA, 3.45 ± 0.82 vs. GA + CA 2.00 ± 0.87, *P* < 0.01), but there were no significant differences between the two groups at 12 or 24 h after operation (*P* > 0.05, see Table 4).

**Table 4:** The mean values of the Face, Legs, Activity, Cry, Consolability (FLACC) scores at different postoperative time points

Total FLACC scores	Group GA mean (± SD) (n = 20)	Group GA + CA mean (± SD) (n = 27)	*p*
1 h	5.35 ± 1.22	3.33 ± 1.00	< 0.01
6 h	3.45 ± 0.82	2.00 ± 0.87	< 0.01
12 h	2.25 ± 0.71	2.07 ± 0.78	0.433
24 h	0.85 ± 0.48	1.04 ± 0.89	0.404

## Discussion

Over the past few decades, with the development and popularization of laparoscopic technology, the laparoscopic-assisted Soave pull-through procedure has become the most widely used operation technique in HD patients, owing to its advantages of minimal invasiveness, quick recovery, and low complications rates [8–10]. Notwithstanding such advantages, the area of surgery includes the abdomen and the anus, which can aggravate the stress reaction during the operation. Pulling out the colon through the muscular sheath is one of the most important and difficult procedures in laparoscopic-assisted Soave pull-through. The anal canal is narrow and the internal sphincter is very weak in infants, making the procedure more difficult. Vigorous anal dilation and excessive anal canal traction during surgery can damage the internal sphincter and have adverse effects on postoperative continence function.Therefore, the ideal anesthesia procedures should provide adequate analgesia and anal muscle relaxation during surgery. However, few studies have focused on the anesthetic procedures during surgery for HD..

General endotracheal anesthesia can cause hemodynamic (53.3% of the case) and respiratory (46.7%) complications during the perioperative period, as well as potential neurotoxicity [11, 12]. The combination of general anesthesia and regional anaesthesia techniques reduces the neurohumoral response to surgery, alleviates intraoperative inhalation and consumption of opioid agents, and accelerates early mobilisation and recovery [13]. Suresh et al. investigation of 18, 650 children who received caudal block showed that the incidence of complications was 1.9% (1.7%—2.1%), demonstrating that the procedure is safe and should be widely used [5, 14]. Therefore general anesthesia combined with caudal block may be the best anesthesia strategy for laparoscopic-assisted Soave pull-through procedure.

The spinal column of children is straight, while epidural adipose tissue, lymphatic vessels, and vascular plexus are abundant, and the sacral canal volume is small. The anesthetic injected into the sacral canal easily spreads to the thoracic epidural space, and the block area can reach the level of 6–8 thoracic vertebrae. The analgesic and muscle relaxant effects of anesthetics not only satisfy the requirements of transanal operation, but also reduce the draw reaction during laparoscopic surgery, and provide more stable hemodynamics. An optimal analgesic effect can avoid the stimulation of the sympathetic adrenal medulla and reduce the release of catecholamine, as well as reduce the irritation caused by tracheal intubation, skin incision and transanal operation. Šabanović Adilović et al. found that caudal block with analgosedation provide better control of intraoperative hemodynamic conditions, postoperative emergence delirium and postoperative pain compared with general endotracheal anaesthesia [15]. In our study, the GA + CA group received general anesthesia combined with caudal block, and the hemodynamic changes during transanal operation were more stable than those in the GA group.

The FLACC scores at 1 h and 6 h after surgery and the mean dose of sufentanil were lower, suggesting that general anesthesia combined with caudal block can provide better analgesic effect, consistently with Adisa’s finding. The duration of operation, and in particular that of transanal operation of the GA + CA group were significantly shorter, and the mean dose of rocuronium was smaller than that in the GA group, indicating that the caudal block could effectively relax the anal muscles, making it easy to pull out the colon through the muscular sheath, thus greatly reducing the difficulty of the operation and shortening the time required by transanal operation. Caudal block has been used in anorectal surgery [16, 17], and was found to be effective in depressing anal sphincter tone, including maximal resting pressure (MRP) and maximal squeezing pressure (MSP), with the percentage of MSP inhibition being greater than that of MRP [18]. In this study, we could not measure the anal sphincter tone because of the limitations of medical conditions. However, we speculate that the shorter duration of the transanal operation in the GA + CA group is closely related to anal sphincter relaxation after caudal block. Alizadeh’s study [19] indicated that caudal block in addition to general anesthesia had a favorable effect on reducing blood loss during operation, operation duration, and analgesic use, agreement with our findings.

Kim et al. found that caudal block significantly reduced the sevoflurane concentration for a smooth laryngeal mask airway removal in anesthetized children, reduced airway complication and led to faster recovery [20]. In the GA + CA group, the recovery time was shorter, which may be due to the lower mean doses of sufentanil and rocuronium. A previous study found that caudal block could provide a more effective and lasting analgesic effect, but was associated to more side effects than general anesthesia [21]. Another study suggested that caudal block was not associated with postoperative side effects [22]. However, in our study we found no differences in the incidence of complications between the two groups.

This is one of the few studies focusing on the anesthetic procedure for HD surgery. Howere,this analysis was limited by its retrospective nature and the small sample size. The patients were aged 3–6 months, with poor vascular reactivity, and the hemodynamic changes were mainly reflected in changes of HR but not SBP and DBP. Furthermore, anal sphincter tone could not be measured due to the limitation of medical conditions.

## Conclusion

We have shown that general anesthesia combined with caudal block can shorten the duration of operation, provide more stable intraoperative hemodynamics, and better postoperative analgesia. In view of the limitations of this study, the role of caudal block needs to be confirmed in a future prospective randomized study.

## Data Availability

The datasets used and/or analyzed during the current study are available from the corresponding author (LX Zhang; dr_l.x.zhang@hotmail.com) on reasonable request.

## Abbreviations

HD: Hirschsprung disease
CA: Caudal blocks
GA: General anesthesia
MAC: Minimum alveolar concentration
HR: Heart rate
SBP: Systolic blood pressure
DBP: Diastolic Blood Pressure
FLACC: Face, Legs, Activity, Cry, Consolability
PIP: Peak inspiratory pressure
MRP: Maximal resting pressure
MSP: Maximal squeezing pressure
